# Telomere Length: Implications for Atherogenesis

**DOI:** 10.1007/s11883-023-01082-6

**Published:** 2023-01-23

**Authors:** Hao Yin, J. Geoffrey Pickering

**Affiliations:** 1 Robarts Research Institute, Schulich School of Medicine and Dentistry, Western University, London, Canada; 2 Robarts Research Institute, Departments of Medicine, Biochemistry, and Medical Biophysics, Schulich School of Medicine and Dentistry, Western University, London, Canada; 3 London Health Sciences Centre, London, Canada

**Keywords:** Telomere length, Atherosclerosis, Coronary disease

## Abstract

**Purpose of Review:**

The purpose of the study is to explore the evidence linking telomere length with atherosclerotic ischemic disease.

**Recent Findings:**

There has been a recent expansion in strategies for measuring telomere length, including analyzing genome sequence data and capitalizing on genomic loci that associate with telomere length. These, together with more established approaches, have been used to generate a more complete picture of telomere length relationships with ischemic disease. Whereas earlier meta-analyses suggested an association between short leukocyte telomeres and ischemic disease, several recent large population studies now provide particularly compelling data, including an association with cardiovascular mortality. In addition, whether short leukocyte telomeres might be causally related to ischemic disease has been interrogated using Mendelian randomization strategies, which point to shorter leukocyte telomeres as a determining risk factor. Importantly however, the wide, interindividual variability in telomere length still means that a single assessment of leukocyte telomere length in an individual does not reliably report on a biological aging process. In this regard, recent multi-tissue analyses of telomere length dynamics are providing both new mechanistic insights into how telomere length and shortening rates may participate in atherogenesis and risk prediction opportunities.

**Summary:**

The balance of evidence indicates that short leukocyte telomeres confer a risk for atherosclerotic cardiovascular disease. Moreover, an integrated analysis of telomere lengths in leukocytes and other tissues may provide a window into individualized telomere dynamics, raising new prospects for risk management.

## Introduction

Telomeres are protein-bound stretches of DNA at the ends of linear chromosomes that protect the ends from damage and prevent them from fusing with each other [1, 2]. A six-nucleotide repeat (TTAGGG) is the sequence hallmark of telomeres, with hundreds to thousands of tandem repeats in human cells [3]. Research on telomeres dates to the late 1930s, but the modern era of telomere research was ushered in with the seminal work of Elizabeth Blackburn, Carol Greider, and Jack Szostak, who elucidated the protective actions of the telomere caps and the enzyme, telomerase, that catalyzes the formation of these caps. Their work was the subject of the 2009 Nobel Prize in Physiology or Medicine [4].

Telomeres provide a solution to what has been termed the “end-replication problem” wherein the 3′ end of the DNA strand is not fully replicated during mitosis. In germ cells and other stem cells, the repeated telomere shortening that would otherwise occur is buffered by the telomerase-catalyzed addition of telomere repeats. The situation is different for somatic cells where there is little to no telomerase activity [5] and thus telomeres progressively shorten with each cell replication. However, adverse consequences of chromosome shortening will be delayed by virtue of the long telomere segments that accommodate the shortening. That said, once a critically short telomere length is reached, the cell is at considerable risk for further genomic stability, and the short telomere can trigger the cell to enter a state of senescence. Cellular senescence, in turn, can be a destructive force for vascular tissues, even if the number of senescent cells is modest. If not cleared by the immune system, senescent cells can secrete a collection of proteins, the senescence-associated secretory profile (SASP), that pathologically modify the local environment [6–9].

Based on these considerations, paradigms pertaining to telomere shortening and chronic, age-associated diseases have been proposed. One paradigm is the possibility that the length of telomeres might serve as a biological aging clock. In adult circulating leukocytes, telomeres shorten at an average rate of about 20–40 bp/y [10–12]. With chronic disease-associated inflammation, cumulative leukocyte shortening can be expected to increase as leukocytes or their progenitors turn over. Moreover, oxidative stress can accelerate telomere loss, at least in vitro [13, 14]. Given this, there has been a prevailing notion that leukocyte telomere length may be a biomarker of biological aging, effectively reporting on the aging process, over and above the chronological age.

A second paradigm is the potential for short telomeres to functionally contribute to diseases of aging. For example, by triggering cell senescence, a cascade of inflammation, extracellular matrix disruption, and cell death pathways might be initiated and/or sustained. Telomere shortening as a functional driver of chronic diseases is a tantalizing hypothesis and, if confirmed to be dominant, could open therapeutic avenues.

Importantly, the extent to which either of these paradigms are valid, and might translate to patient care, remain topics of inquiry. In this review, we summarize recent data pertaining to these conceptual telomere length paradigms with a focus on atherogenesis and ischemic vascular disease. We first review the diverse technologies employed for assessing telomere length, including newer approaches that have advanced the field. We then highlight recent studies investigating associations between leukocyte telomere length and cardiovascular disease and summarize data addressing the question of causality. We also discuss the limitations in considering leukocyte telomere length as an indicator of biological aging, and we review refined hypotheses that are emerging from studies comparing the length of telomeres in leukocytes with that in other tissues.

## Measuring Telomere Length for Atherosclerosis Research

Assessing the length of chromosome ends is technically more challenging than quantifying many of the existing and potential biomarkers of atherosclerosis. As such, developing new methods has been an active area of investigation. Southern blot analysis stands as the classic, robust approach for assessing telomere length. Although some sub-telomeric DNA will be included in this measurement, the approach provides an absolute measure of telomere length (i.e., in kilobases) with relatively small measurement errors [15, 16]. However, Southern blotting requires microgram amounts of genomic DNA. This has made polymerase chain reaction (PCR) approaches attractive and widely used. The basic PCR strategy entails ascertaining the copy number ratios of telomere repeats to a single-copy gene [17]. Measurement error may be higher than with Southern blot analysis [18], but it is a cost-effective strategy needing small amounts of input DNA and with the ability to scale to high-throughput studies. An important example of this is data from the UK Biobank, in which over 450,000 individuals have had leukocyte telomere assessments undertaken using multiplex quantitative PCR [19•].

A hybridization approach has also recently been developed as a high-throughput strategy for relative telomere length assessment [20, 21]. This approach also requires only nanogram quantities of input DNA but does not entail DNA amplification. Instead, a telomere repeat sequence signal and reference gene signal are amplified using branched DNA technology and detected using a Luminex, bead-based technology. This approach has recently been used to evaluate over 6000 samples from donors in the Genotype-Tissue Expression (GTEx) project [22••].

Importantly, the above approaches measure average telomere length from a bulk genomic DNA harvest. Such an assessment does not allow for determining chromosome-specific or cell-specific telomere lengths nor does it enable gauging the shortest telomere length in a sample. Chromosome-specific telomere length can be assessed using single telomere length analysis (STELA), which uses a combination of hybridization, PCR amplification with chromosome-specific primers, and Southern blot analysis [23]. More recently, a ligation and PCR-based telomere shortest length assay (TeSLA) has been developed that reliably measures telomere length in all chromosomes and the distribution of the shortest telomeres [24, 25]. Telomere length can also be assessed in single cells using quantitative fluorescence in situ hybridization (FISH) together with either flow cytometry (flow-FISH) or in situ analysis of metaphase cells, standard fixed (interphase) cells, or tissue sections (Q-FISH) [26–29]. These strategies have the potential to relate telomere lengths with single-cell phenotypes. To our knowledge, high spatial resolution assessment of telomere length in atherosclerotic tissue has not been reported. However, such an approach would be important for evaluating if there are culprit cells with short telomeres within the plaque that drive pathology.

Another important advance in telomere length assessment is utilizing whole genome or exome sequencing data. Ascertaining telomere length from sequencing data is an appealing strategy given the conceptual simplicity, the declining costs of genome sequencing, and the large amount of additional sequence data that can be simultaneously ascertained. The challenge of identifying the origin of a given telomere repeat region and aligning this with a reference genome has been now circumvented by bioinformatic tools, including including Motif_counter [30], TelSeq [31], Computel [32], qMotif [33], and Telomerecat [33]. Recent large studies involving > 100,000 individuals from both the NHLBI Trans-Omics for Precision Medicine (TOPMed) program and the UK Biobank have demonstrated the power of this approach of telomere length assessment [34, 35•]. Another important recent example is research implicating clonal hematopoiesis of indeterminate potential in both telomere length and coronary artery disease [35•].

Finally, an exciting but distinct approach to assessing telomere length capitalizes on the fact that telomere length is strongly genetically determined. There is wide inter-individual variation in mean leukocyte telomere length, and heritability is estimated to account for between 44 and 80% of the variance [10, 36, 37]. Growing numbers of genomic loci have been found to associate with telomere length, and although to date these explain only a minority of the variation in the population, analysis of variant associations has been used as an indicator of telomere length [34, 38, 39, 40••]. This analysis identifies the “genetically determined” telomere length, but similarities with PCR-determined telomere length have been reported [39, 40••].

In summary, over a relatively short time frame, there has been tremendous expansion in strategies for assessing telomere length. This diversity has increased the power of telomere length analyses and provides a repertoire of strategies that can be tuned to the specific scientific questions asked.

## Association of Short Leukocyte Telomeres with Atherosclerosis

Because atherosclerosis is an aging-related disease with an accumulated burden of oxidative and inflammatory stresses, there has been great interest in delineating relationships with telomere lengths. Yet, there have been conflicting data, and despite a large body of studies, there are questions regarding the strength of any association, their predicative value, causal relationships, and mechanistic linkages. However, compelling data are now emerging.

An early hint that leukocyte telomere length may be related to atherosclerosis came from a small cross-sectional study that found that mean telomere length in leukocytes was shorter in individuals with 3-vessel coronary disease than those with normal coronary arteries, independent of risk factors for coronary disease [41]. This study included only 10 patients with coronary disease which, given the wide interindividual variations in telomere length, is too small to reliably detect differences. Nonetheless, this is an instance where, rather than proving to be over-enthusiastic reporting, the early small study findings have been borne out by subsequent research. In a meta-analysis of 27 studies evaluating the association of telomere length and cardiovascular outcomes, D’Mello reported significant associations between shorter leukocyte telomere length and myocardial infarction, stroke, and type 2 diabetes [42]. Significant associations with angina or non-fatal ischemic heart disease were not found. Another meta-analysis of 24 studies, with some overlap of studies, found an association between shorter leukocyte telomere length with non-fatal myocardial infarction, coronary vascular disease, and coronary revascularization, but, in contrast with the analysis of D’Mello et al., not clearly cerebrovascular disease [43].

More recent observational data from larger populations have strengthened the evidence for an association between short leukocyte telomeres and ischemic disease. PCR assessment of leukocyte telomere length in 66,618 Danish subjects, 17,235 of whom were diagnosed with ischemic heart disease, revealed that a 200-bp-shorter telomere length was associated with a multivariable adjusted hazard ratio for ischemic heart disease of 1.02 [44]. Although the hazard identified was not high, recent mortality data support the importance of the association. Longitudinal follow-up of 472,432 English participants within the UK Biobank data revealed a modest but highly significant association between reduced leukocyte telomere length and cardiovascular mortality (adjusted hazard ratio 1.09) [19•]. This association with cardiovascular mortality adds strength to previous reports finding either a mortality trend [11] or a significant association specifically in older subjects [45] or via meta-analysis [42]. The UK Biobank data also revealed a modest relationship between shorter leukocyte telomeres and overall mortality (hazard ratio 1.08). Interestingly, mortality from pulmonary, digestive, and musculoskeletal disorders were most positively associated with reduced telomere length [19•].

## Tightening the Association Between Short Telomeres and Atherosclerotic Cardiovascular disease: Consequence, Confounded, or Causal?

Cross-sectional observational studies of telomere length have generally sought to statistically account for known confounding variables, recognizing that many cardiovascular risk factors themselves are associated with shorter telomeres [11]. However, unknown confounders can still complicate the interpretation of any associations identified. Also, there is the possibility that shorter leukocyte telomeres arise as a consequence of ischemic heart disease and any resulting leukocyte or progenitor cell mitotic activity, rather than an upstream determinant. A clue that such “reverse causality” might not underlie the association first came from the West of Scotland Primary Prevention Study [46]. A nested case–control analysis of this primary prevention trial with pravastatin revealed that those with a shorter telomere length at the time of recruitment had a higher risk of developing coronary disease during follow-up. Although this finding did not indicate causality, it supported the possibility that factors contributing to leukocyte telomere shortening might also contribute to the risk of coronary artery disease.

Another approach taken to exploring if short leukocyte telomere length is a determinant of ischemic disease has been to study subclinical atherosclerosis, i.e., atherosclerosis evident by imaging biomarkers in symptom- and event-free individuals. If short telomeres are functionally linked to atherosclerosis development, then one would expect short telomeres in those with pre-clinical atherosclerosis. Notably, however, two studies that took this approach did not find such a relationship. Among 2509 subjects in the Asklepios Study cohort, leukocyte telomere length as assessed by Southern blot analysis was not found to be an independent predictor of intima-medial thickness or plaque presence in the carotid and femoral arteries [47]. Fernandez et al. undertook a cross-sectional analysis of 1459 individuals from the PESA (Progression of Early Subclinical Atherosclerosis) study in whom both mean circulating leukocyte telomere length and the proportion of leukocytes with short telomeres (< 3 kb) were evaluated using fluorescent in situ hybridization. Neither the average telomere length nor the short telomere load were found independently associate with markers of subclinical atherosclerosis, including coronary calcium content and ultrasound assessment of carotid and femoral artery plaque burden [48].

Although these studies of pre-clinical atherosclerosis did not support a determinant relationship between leukocyte telomere length and ischemic disease, a number of recent large population genetics studies have re-ignited the question and yielded compelling evidence that individuals with short leukocyte telomeres are indeed predisposed to ischemic heart disease. Using genome-wide association analysis, Codd et al. identified seven loci (five new) with genetic variants that affect mean telomere length, and a weighted genetic risk score analysis of 22,233 subjects in the CARDIoGRAM cohort revealed the combined variants were associated with increased risk of coronary disease [38]. Additionally, recent studies using a Mendelian randomization design have been important, recognizing that this approach is less impacted by residual confounding variables and also avoids the conflation of consequence and cause. Using three genetic variants associated with shorter telomere length (different from the above seven) as instrument variables for Mendelian randomization analysis and 184,967 subjects from the CARDIoGRAMplusC4D consortium, Scheller Madrid found that a genetically determined 200 bp-shorter telomere length was associated with a statistically significant odds ratio of 1.10 for ischemic heart disease [44].

Recently, further analysis of the UK Biobank identified 197 genetic variants that explained 4.54% of the variance in leukocyte telomere length, and Mendelian randomization also established an association between genetically determined leukocyte telomere length and coronary artery disease. This association between shorter genetically determined telomere length and higher risk of coronary disease was not found to be explained by blood pressure (which in fact associated with high genetically determined telomere length) or plasma lipid profiles. Lower life expectancy was also estimated for those who, at age 40, had a leukocyte telomere length one standard deviation below the mean by about 2.5 years compared to those with leukocyte telomere length one standard deviation above the mean [40••].

Some genetic/epidemiologic studies have begun to address potential mechanistic elements in the path between short telomeres and ischemic disease. Using a network Mendelian randomization approach, with statistics from several genome-wide association studies, Zhan and coworkers not only found an association between genetically determined telomere length and coronary heart disease but also identified that fasting insulin was causally associated with genetically determined telomere length [49]. Mediation analysis revealed that increased fasting insulin could be positioned within a pathway from short telomere length and ischemic heart disease. Although mechanistic links between short telomere length and insulin resistance are unknown, the case for shortened telomeres contributing to ischemic heart disease is strengthened by a potential mediating mechanism.

Another intriguing potential mediator between short telomere length and ischemic heart disease is clonal hematopoiesis of indeterminate potential (CHIP). CHIP is a new risk factor for coronary artery disease [50, 51] and cross-sectional analyses have shown that CHIP correlates with leukocyte telomere length [52]. Recent Mendelian randomization studies have found a rather complex interplay with coronary disease. Longer telomere length was found to be a promoting factor for the acquisition of CHIP, whereas CHIP itself contributed to telomere shortening. Mediation analysis further identified that the CHIP-associated coronary disease risk was, in small part, driven by the shortened leukocyte telomere length.

The balance of evidence therefore points to short leukocyte telomere length being not only associated with ischemic disease but a risk factor for the disease. The associations appear relatively weak, although this is possibly because assessing average leukocyte telomere length dilutes the linkages between telomere length and ischemic disease. Further studies on the burden of select cells with critically short, senescence-inducing telomeres, either in the circulation or the vessel wall, as well as assessing telomere dysfunction independent of length, could provide further valuable advances [53–55]. Furthermore, the genetic tools for analyzing telomere length variation will only get stronger as more genetic determinants are identified.

## Insights from Telomere Length Assessments Among Different Tissues

Although leukocytes have been a valuable and convenient source for assessing telomere length, it is important to recognize that the length of telomeres in adult leukocytes differs from that in other tissues. Differences in accumulated cell replication profiles, tissue regenerative capacity, and the size of the local stem pool result in different telomere lengths among adult tissues [22••]. For example, compared to telomeres in circulating leukocytes, those in skeletal muscle can be 70% longer, with telomeres in reproductive organs being even longer [22••, 56••]. This does not negate the value of measuring leukocyte telomere length — genetic variants known to affect leukocyte telomere length also affect the length of telomeres in other tissues — and the variance in telomere length among tissues within an individual is less than the variance between individuals [22••]. As well, it has emerged that telomeres in many tissues shorten with age, as they do in leukocytes [22••, 56••, 57]. However, differences in both telomere length and telomere shortening rates can be substantial. Recently, new insights have emerged by capitalizing on these differences.

**Fig. 1:**
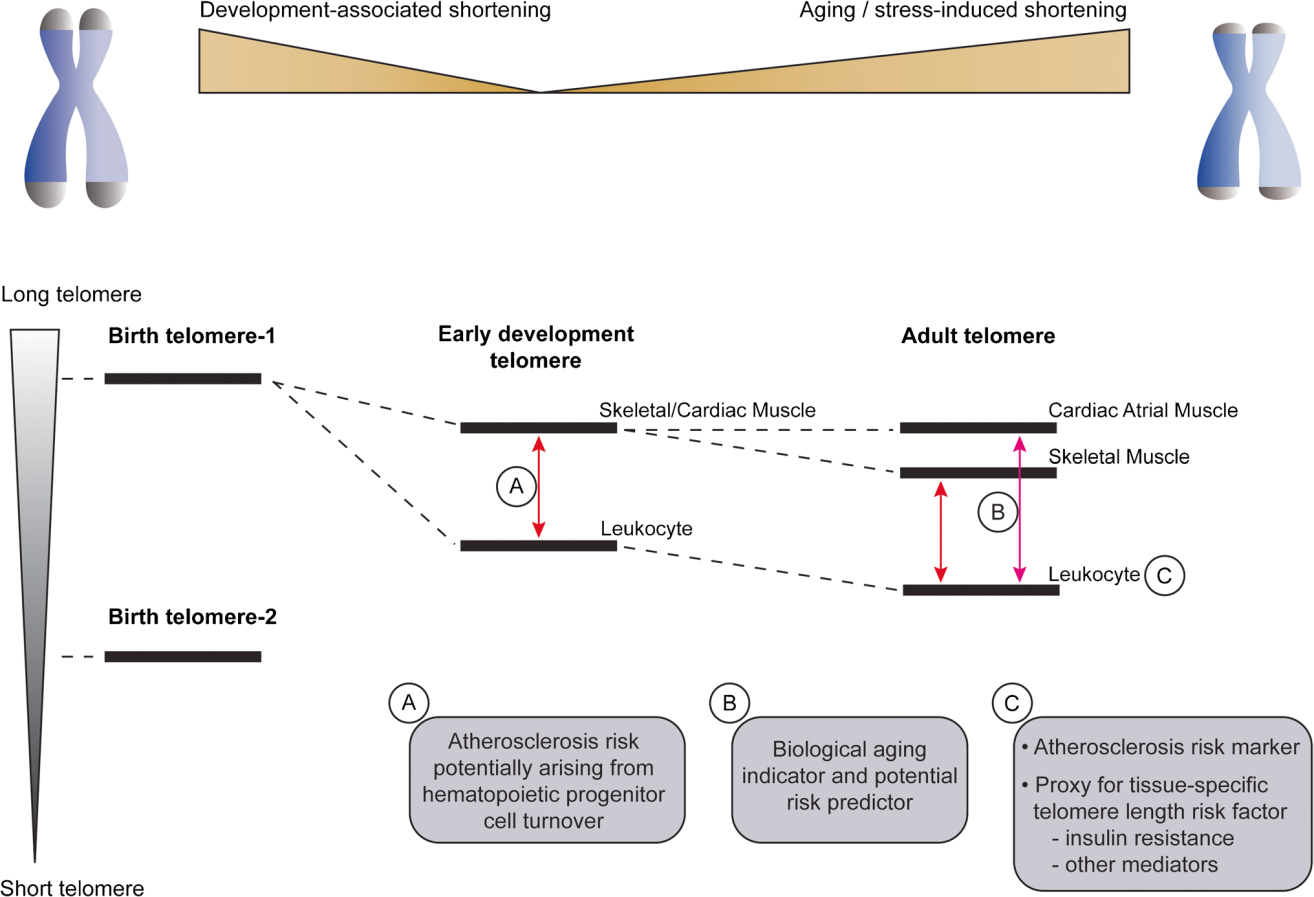
Telomere length dynamics and atherosclerosis. There is wide genetically determined inter-individual variation in telomere length, reflected in the two birth telomeres depicted. Telomeres shorten during early development, when cell replication is high, and throughout adulthood depending on the replicative and oxidative stress burdens. Telomere shortening during early development is greatest in peripheral blood leukocytes, producing a telomere length gap with somatic tissue telomeres, including in skeletal and cardiac muscle (**A**). During adulthood, telomere attrition in skeletal muscle is similar to that in leukocytes but telomere length in cardiac atrial muscle is relatively stable over six decades (**B**). Short telomeres in adult circulating leukocytes, which will reflect relatively short telomeres in somatic tissues, are associated with and confer atherosclerosis risk (**C**). Insulin resistance is one proposed mediator. The patient-specific clinical value of a single leukocyte telomere length assessment remains to be determined. Individuals with wide muscle-leukocyte telomere length difference are predisposed to atherosclerotic disease (**A**), and the cardiac atrial muscle-leukocyte telomere gap may also capture stress accumulation and biological aging, with potential risk prediction value (**B**)

The idea that a single leukocyte telomere length assessment will serve as a biological aging clock, and potentially a read-out of the accumulated risks for atherosclerosis, is not consistent with what is currently understood regarding telomere length dynamics. Leukocyte telomere length reflects a combination of birth telomere length, which varies widely in the population, and the subsequent accumulated telomere shortening in leukocytes or their progenitors (Fig. 1). Leukocyte telomeres will shorten in response to replicative and oxidative stresses, but, because inheritance is a substantial determinant of adult telomere length [10, 38, 58], a single measure of adult leukocyte telomere length will not inform on the telomere shortening rate. However, telomeres throughout many body compartments do shorten, during early development when cellular replication is abundant and thereafter depending on replicative and oxidative stresses imposed on the specific tissue. For the latter, 5–10 years can pass before a change is detectable making serial measurements impractical [59, 60].

In dogs, it was found that leukocyte telomere length was only modestly age-related, but the difference in telomere length between skeletal muscle and leukocytes was substantially age-related. This suggested that the skeletal muscle-leukocyte telomere length difference afforded pseudo-longitudinal data on leukocyte telomere attrition post early-development [61]. In humans, the scenario is not as clear cut. Although adult skeletal muscle telomeres are much longer than those in leukocytes, the telomere shortening rates in adults have been found to be similar in both compartments [56••, 57]. Interestingly however, the difference between skeletal muscle and leukocyte telomere lengths has been found to be wider in individuals with atherosclerotic vascular disease than in control subjects without vascular disease [62••]. Because much of the skeletal muscle-leukocyte telomere length difference will arise during the first decades of life, including with hematopoietic stem cell expansion, it is proposed that the increased telomere length gap in those with vascular disease precedes the development of atherosclerosis. That is, leukocyte telomere attrition, particularly early, underlies the shorter telomere length in individuals with atherosclerotic vascular disease. It is further proposed that hematopoietic stem cell turnover, and its linkage to endothelial cell progenitors, may be at play [63] (Fig. 1).

However, not all tissues will have a telomere shortening rate in the adult as fast as in leukocytes. In cross-sectional analyses, we found, for example, that whereas skeletal muscle telomere length declined with age similar to leukocyte telomere length, cardiac atrial tissue telomere length was stable over six decades of life [56••]. Thus, the cardiac muscle-leukocyte gap captures the spread in the early life but also with the aging- and disease-related stresses in adult life. Importantly, we found that among patients undergoing cardiac surgery, the cardiac-leukocyte telomere length difference predicted adverse in-hospital outcomes [56••]. In contrast, the skeletal muscle-leukocyte telomere gap and leukocyte telomere length itself did not predict outcomes.

These refined multi-tissue analyses of telomere length dynamics with internal referencing add a new dimension to untangling associations with atherosclerosis and provide evidence that telomere assessments may have predictive value for clinical care. This is important because the value of ascertaining telomere length data on an individual for clinical decision-making has otherwise been quite uncertain [48, 64]. Bringing a telomere assessment to the individual patient level thus remains an exciting prospect, but additional studies are warranted.

## Conclusions

The balance of evidence, including from recent large genetics-based analyses, indicates that short leukocyte telomeres are a risk factor for ischemic heart disease. Short telomeres are not only associated with ischemic heart disease but appear to have a contributory role, albeit not necessarily a dominant one. However, caution is needed with adopting the notion that a single assessment of leukocyte telomere length can meaningfully serve as a personal biological aging clock. This is because of wide inter-individual variability in telomere length that is substantially genetically determined. Recent studies evaluating the difference in length between non-leukocyte telomeres and leukocyte telomeres have shed new insights into pathobiology of ischemic heart disease and can provide a window into individualized telomere dynamics and potentially personalized risk. Future studies on genetic determinants of telomere length and dysfunction, and on plaque cell-specific telomere dynamics in situ, will further refine our understanding of atherosclerosis development and events.
